# Necrotizing Enterocolitis: What’s New and What’s Next?

**DOI:** 10.3390/ijms26199660

**Published:** 2025-10-03

**Authors:** Cuilee Sha, William R. Sander, Kathryn Bass, Helen Hsieh, Agnieszka B. Bialkowska

**Affiliations:** 1 Medical Scientist Training Program, Renaissance School of Medicine at Stony Brook University, Stony Brook, NY 11794, USA; 2 Department of Surgery, Renaissance School of Medicine at Stony Brook University, Stony Brook, NY 11794, USAhelen.hsieh@stonybrookmedicine.edu (H.H.); 3 Center for Nervous System Disorders, Stony Brook University, Stony Brook, NY 11794, USA; 4 Department of Medicine, Renaissance School of Medicine at Stony Brook University, Stony Brook, NY 11794, USA

**Keywords:** necrotizing enterocolitis, inflammation, biomarkers, disease models

## Abstract

Necrotizing enterocolitis (NEC) is a significant cause of morbidity and mortality for preterm infants in the neonatal intensive care unit. From the first surgical approaches for NEC in the 1970s and the development of Bell’s staging criteria, there has been a continuous medical and scientific journey towards understanding the pathophysiology, clinical progression, and treatment possibilities for this devastating disease. Basic science research has played a crucial role in understanding the pathogenesis of NEC. In vivo NEC models, which include rodents (mice, rats) and pigs, and in vitro NEC models, which utilize intestinal cell lines and organoids, have identified critical disease biomarkers, pathways in NEC pathogenesis, and novel therapeutic targets. These potential therapies have been brought into clinical trials to improve treatment options for infants with NEC. This review will provide a comprehensive assessment of research conducted over the last decade, leading to a deeper understanding of the disease’s development and progression through the use of innovative models, the identification of novel biomarkers, the development of new therapeutic approaches, and, finally, an overview of the latest clinical trials. We will conclude with a discussion of ongoing challenges and future research directions, highlighting the optimism and hope that these advancements bring to the field of neonatology and pediatric surgery. This review will serve as a reference and guide for future NEC research, with the ultimate goal of enhancing clinical outcomes and improving the quality of life for patients with NEC and their families.

## 1. Introduction

Necrotizing enterocolitis (NEC) is one of the most destructive gastrointestinal diseases affecting newborns, especially infants born prematurely or with very low birth weight [1,2,3]. Characterized by inflammation and necrosis of the intestinal wall, NEC can progress rapidly, leading to intestinal perforation, sepsis, and in severe cases, death [1,3,4]. Despite extensive research, the pathophysiology underlying NEC is still incompletely understood, which complicates efforts in its prevention, early diagnosis, and treatment. Several hypotheses have been proposed to explain the pathogenesis of NEC. A central theory suggests that an immature immune system, when confronted with a pathogen, responds with an uncontrolled inflammatory immune response, which, inappropriately, progresses to sepsis [2]. Overexpression of toll-like receptor 4 (TLR4) in immature immune cells has also been implicated as a potential causative factor, leading to an upregulation of pro-inflammatory mediators [2,5]. Other proposed mechanisms include impaired intestinal mucosal repair, which decreases blood perfusion and results in bowel ischemia, necrosis, and dysbiosis or abnormal microbial colonization of the neonatal gut [2,6].

We conducted a comprehensive search in PubMed using Medical Subject Headings (MeSH) and corresponding free words, with a primary focus on basic, translational, and clinical research published in the last fifteen years to highlight the most recent advances in the field of NEC. The major search terms are as follows: “necrotizing enterocolitis”, “epidemiology”, “clinical score”, “pathophysiology”, “diagnosis”, “management”, “clinical trials”, “biomarkers”, “inflammatory biomarkers”, “fecal biomarkers”, “metabolomics and metagenomics-based markers”, “proteomics biomarkers”, “genomic-based biomarkers”, “animal models”, “in vitro”, “in vivo”, “organoids,”, and “management”. The above search terms were linked by the logical operators “OR” or “AND.” A total of 9109 references were retrieved. We excluded studies that were old (unless the study was foundational to the field), repetitive, non-English, or lacked clear information and selected a number of representative scientific papers. Ultimately 164 references were included in the final manuscript.

## 2. Pathophysiology of NEC

### 2.1. Epidemiology and Risk Factors

The mortality rate for NEC is as high as 50%, which makes it major cause of death in preterm infants [4,7]. Nearly 70% of cases occur in preterm infants born before 36 weeks of gestation; NEC accounts for almost 8% of all neonatal intensive care unit (NICU) admissions [4]. Moreover, the incidence of NEC increases fivefold for infants with extremely low birthweight (<1000 g) or born extremely early (<28 weeks of gestation) [3]. Although most cases of NEC occur in preterm infants, 10% of cases are seen in term infants [8]. Major risk factors affecting susceptibility to NEC include prematurity, abnormal bacterial colonization of the gut, and formula feeding [9,10,11]. Additional factors associated with an increased incidence of NEC include mechanical ventilation, congenital defects, and a low Apgar score [11,12,13,14].

### 2.2. Clinical Presentation and Diagnosis

The clinical presentation of NEC is highly variable. While early symptoms are often nonspecific, disease progression follows certain patterns. Typically, NEC presents with a sudden onset of feeding intolerance, abdominal distension, bloody stools, and nonspecific systemic changes a couple of weeks after birth [1,15].

**Figure 1:**
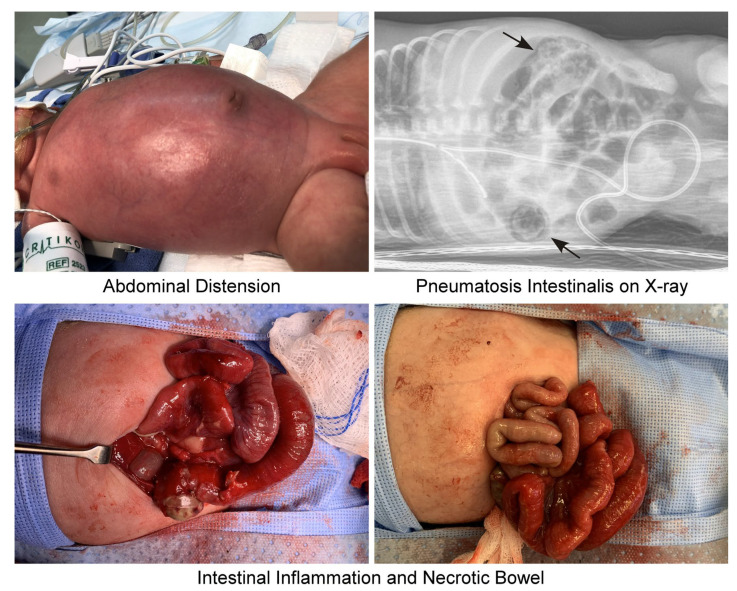
**Clinical symptoms of NEC.** NEC presents with a variety of clinical and radiological signs, requiring the Bell’s staging criteria to guide treatment and management of the disease. Earlier findings include abdominal distension (top left) and bloody stool. On X-ray, NEC presents with pneumatosis intestinalis or gas in the intestinal wall (top right, black arrows). Worsening of the disease, during which surgical intervention would be required, involves perforated intestinal damage and necrotic bowel tissue (bottom). Images were taken at Stony Brook Medicine by Helen Hsieh (author) and de-identified.

Clinical staging of NEC uses the modified Bell’s scale, which classifies NEC into three stages based on clinical and radiological signs (Table 1) [16,17,18]. Stage I of Bell’s criteria includes patients with only mild gastrointestinal disturbances and normal to mild intestinal dilation on radiologic imaging [16]. Nonspecific systemic signs, including temperature instability, tachycardia, bradycardia, apnea, and lethargy, are seen at this stage [16]. In the second Bell’s stage, patients present with worsening systemic signs, now including mild metabolic acidosis and thrombocytopenia [16]. Additional indications include abdominal distension and tenderness, absent bowel sounds and pneumatosis intestinalis seen on imaging [16]. The third stage of Bell’s criteria encompasses patients with worsening stage II symptoms as well as hypotension, disseminated intravascular coagulation, intestinal perforation in severe cases, and pneumoperitoneum [16]. Patients with intestinal perforation require emergent surgery, thus significantly increasing the mortality rate of NEC. These clinical findings are shown in Figure 1. Of note, stage I of the Bell’s Criteria is considered “suspected” NEC whereas stages II and III are termed “definite” and “advanced” NEC, respectively [16].

### 2.3. Long-Term Implications

Survivors of NEC often face a range of long-term complications that extend beyond the scope of gastrointestinal health. These complications depend on the severity of the initial episode of NEC, the extent of bowel involved, and whether surgical intervention was required. Serious gastrointestinal sequelae of NEC include strictures, intestinal failure, small bowel obstruction, and short bowel syndrome [18,19,20]. Additional concerns of NEC include serious neurological and neurodevelopmental consequences resulting from sustained inflammation [18,19]. These complications include intraventricular hemorrhage, periventricular leukomalacia, and white matter injury, which can be indicative of further long-term injury [19]. Reduction in quality of life can severely impact mental health and social experiences had by NEC patients and their families [19]. Patients and families frequently report ongoing challenges in accessing appropriate follow-up care and support. This underscores the long-term burden of the disease and the need for standardized follow-up and assessments [19].

Given the significant mortality and morbidity as well as gaps in effective preventative and therapeutic strategies, a comprehensive understanding of NEC remains critically important. This review aims to provide an updated collection of current knowledge on the clinical features and emerging strategies in the management and prevention of NEC.

## 3. Current Treatment Options and Novel Approaches

### 3.1. Management of NEC

**Figure 2:**
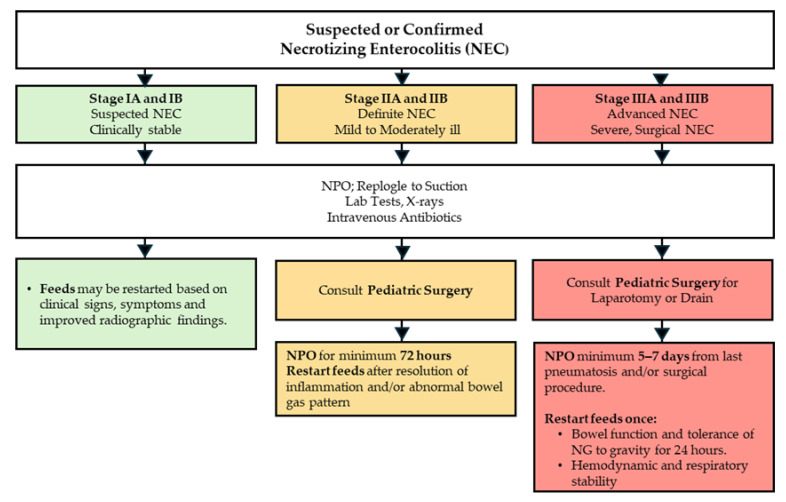
**Management of NEC.** All infants with NEC are put on bowel rest and kept NPO, monitored with lab tests and X-rays, and treated with intravenous antibiotics. The severity of the disease determines further management. Stage I NEC patients may have feeds restored if they show clinical improvement. Stage II NEC patients must be NPO for at least 72 h and can only have feeds restored if they no longer have inflammation or abnormal bowel gas patterns. It is also recommended to consult pediatric surgery for these patients. Stage III NEC patients require a pediatric surgery consult if they need surgical treatment with either a laparotomy or a drain. Additionally, these patients must be NPO for at least 5–7 days and can only have feeds restored if bowel function returns for at least 24 h, and they are systemically stable.

Current treatment options for NEC are unfortunately limited and, to some extent, depend on the clinical status of NEC. For mild, suspected, and moderate NEC, infants are kept without any nutrition by mouth (NPO), provided total parenteral nutrition (TPN) and intravenous antibiotics in a monitored clinical setting. Additionally, a nasogastric tube (NGT) is placed to relieve abdominal distension by removing air and fluid from the gastrointestinal tract. Frequent abdominal X-rays and laboratory tests monitor for patient stability and determine disease progression. In the setting of bowel necrosis and perforation or in the event of failure of medical management, surgical interventions, such as drain placement or laparotomy, are considered (Figure 2) [16,18,21,22,23].

### 3.2. Clinical Trial Landscape in NEC

A thorough examination of the completed, ongoing, and planned clinical trials best represents the current landscape of NEC-based clinical research. Clinical trial data were aggregated from the National Library of Medicine and the Eunice Kennedy Shriver National Institute of Child Health and Human Development using the key search term “Necrotizing Enterocolitis”. Output data (n = 79 trials) were manually categorized by study type (Drug Therapy, Non-surgical, Dietary Supplement, Observational, Antimicrobial Therapy), and further examined based on NEC outcome, Focus, Study Status, and Study Results (Table 2, search performed on 10 June 2025). While many trials do not directly examine NEC, nearly all examine NEC as a primary or secondary outcome and all trials focus on preterm delivery, a key risk factor for NEC.

#### 3.2.1. Drug Therapy

Drug therapy represented the largest category of clinical trials, comprising 28 studies in our collected dataset. NEC was designated as a primary outcome in 7.1% of these trials, a secondary outcome in 85.7%, and another outcome in 7.1%. The majority of studies focused on neonates (64.3%), while maternal involvement comprised the rest (35.7%). Most trials were completed (60.7%), and 46.4% of all drug therapy trials had reported results. This category includes a wide range of therapeutics administered to either neonates or mothers. Neonatal interventions included (1) a Phase 3 trial evaluating gut-protective agents such as vitamin A and enteral glutamine in neonates (NCT00707785), (2) a Phase 2 trial in intubated preterm infants evaluating anti-inflammatory and immunomodulatory agents like CC10 protein and immune globulin (NCT01473264), (3) a Phase 3 trial in low birth weight infants investigated non-steroidal anti-inflammatory drugs such as indomethacin (NCT00009646), (4) a Phase 3 trial, designated the Preterm Erythropoietin Neuroprotection Trial (PENUT), studied hematopoietic agents including recombinant human erythropoietin (NCT01378273) [24], and (5) trials investigating neuroprotective agents such as caffeine and inositol (NCT00809055, NCT06448780, NCT06855108, NCT01954082). Trials involving maternal therapies included investigations into CMV hyperimmune globulin (NCT01376778), hormone and endocrine therapies such as progesterone (NCT00615550), 17-hydroxyprogesterone (NCT03292731, NCT00439374, NCT00439374, and NCT00099164), and levothyroxine (NCT00388297), as well as cardiovascular and antithrombotic agents including aspirin (NCT06980025) and pravastatin (NCT03944512). Although all of these drug therapy studies evaluated NEC as a primary or secondary outcome, none reported a statistically significant improvement in NEC outcomes, emphasizing the need for the development of effective pharmaceutical options in the treatment of NEC.

#### 3.2.2. Non-Surgical Interventions

Non-surgical interventions, comprising 14 studies, focused exclusively on neonates. NEC was a primary outcome in 14.3% of these trials, a secondary outcome in 71.4%, and another outcome in 14.3%. Completed studies comprised 57.1% of the category, and 35.7% had reported results. These trials investigated a range of neonatal procedures, including delayed cord clamping to improve hemodynamic stability and placental transfusion (NCT00840983, NCT01866982, and NCT04413097), respiratory support strategies aimed at reducing neonatal respiratory distress syndrome (NCT05446272), transfusion thresholds designed to prevent NEC (NCT06676904), feeding practices examining the route and timing of nutritional administration (NCT01203475), and phototherapy studies exploring the role of bilirubin conjugation in NEC development (NCT00114543). Among the completed trials, several reported lower incidences of NEC. Specifically, a study of inhaled nitric oxide in preterm infants not requiring mechanical ventilation (NCT00955487) found NEC rates of 8.5% in the treatment group versus 15.4% in the control group; however, the difference was not statistically significant (RR = 0.54; 95% CI: 0.20–1.49) [25]. A trial evaluating feeding tube dwell time (NCT03728608) reported NEC in 5.3% of infants with short dwell times compared to 8.0% with longer dwell times. Additionally, a study of gastric aspiration practices (NCT01863043) found fewer episodes of NEC in infants who did not undergo routine aspiration prior to feeding; however, this reduction was not statistically significant.

Many of these non-surgical interventions, though not explicitly designed to reduce NEC, may reduce the risk of NEC. With follow-up studies, these interventions show promise for advanced personalized neonatal care and reducing the occurrence of NEC.

#### 3.2.3. Dietary Supplements

Dietary supplementation was the primary focus of 10 studies within our collected dataset, with NEC as a primary outcome in 20%, secondary in 60%, and other in 20%. Neonates were the primary focus in 70% of these trials, with maternal involvement in 20% and paternal in 10%. Completed studies accounted for 60% of the category, while 40% had reported results. These trials included neonatal micronutrient supplementation such as high-dose vitamin D (NCT05459298), omega-3 and omega-6 fatty acids (NCT00135902), and manganese in fortified parenteral nutrition (NCT00392977); however, none reported significant results. Other studies have examined milk feeding strategies, including comparisons between donor milk and formula (NCT00005888 and NCT01534481), and the feasibility of initiating early feeds in neonates with gastroschisis (NCT06878950). In one of the milk feeding strategy studies (NCT01534481), a statistically significant reduction in occurrence of NEC was observed in infants fed donor human milk as opposed to preterm formula [26]. Maternal supplementation trials focused on antioxidants such as vitamins C and E (NCT00135707) and omega-3 fatty acids (NCT00135902), while the single paternal study investigated folic acid and zinc supplementation (NCT01857310). These maternal and paternal-centered trials did not yield any significant reductions in NEC incidence. Several recent systematic reviews provided in-depth evaluations of the latest clinical trials investigating the possibility of probiotics in the prevention and treatment of NEC. In summary, treatment with probiotics showed a trend of improvement in reducing NEC and mortality in all cases [27,28,29,30,31,32].

#### 3.2.4. Observational Studies

Observational studies comprised 8 trials, with 50% of trials noting NEC as a primary outcome and 50% as a secondary outcome. Neonates were the focus in 75% of these studies, with maternal populations represented in 25%. A large majority of these trials were completed (87.5%); however, none had reported results yet. These studies explored biomarker identification, including epidermal growth factor (NCT00059449) and inflammatory cytokines associated with NEC (NCT01035697), surgical outcomes and complications related to NEC procedures, aspects of premature neonatal development such as respiratory control and growth (NCT03174301 and NCT01203475), and maternal and perinatal risk factors, including morbidity during the COVID-19 pandemic and the establishment of a perinatal biobank (NCT04519502 and NCT00339235). None of these studies has reported statistically significant changes in NEC outcomes.

#### 3.2.5. Antimicrobial Therapy

Antimicrobial therapy was investigated in 8 studies, with NEC designated as a primary outcome in 50%, secondary in 37.5%, and other in 12.5%. The majority of studies focused on neonates (75%), with maternal involvement in 25% of cases. Most trials were completed (75%), of which 75% had reported results. These studies explored both neonatal and maternal antimicrobial strategies in relation to NEC.

Neonatal-focused trials primarily examined how antibiotic exposure affects the microbiome and the risk of NEC. A randomized study of preemptive antibiotic use in infants <33 weeks gestation (NCT02784821) found a significantly higher NEC incidence in infants given antibiotics despite low infectious risk (27.1%) compared to those randomized to no antibiotics (13.7%), with no NEC cases in infants for whom antibiotics were not indicated. A pharmacokinetic study of metronidazole in premature infants (NCT01222585) demonstrated that drug clearance increases with postmenstrual age, informing age-adjusted dosing strategies.

Maternal antimicrobial studies focused on prophylactic antibiotic administration during delivery, particularly in the context of cesarean section. These trials evaluated whether maternal treatment could reduce neonatal infection rates and mitigate prematurity-associated complications, including NEC. One such study shows that treatment with azithromycin reduced maternal infection; it had no significant impact on neonatal outcomes (NCT01235546) [33].

#### 3.2.6. Surgical Interventions

Surgical interventions were the subject of 3 studies. NEC was a primary outcome in 2 trials and a secondary outcome in one. All studies were completed, and results were reported in 2 studies. These trials included comparisons of surgical approaches, such as primary peritoneal drainage versus laparotomy for NEC treatment (NCT00252681), spontaneous intestinal perforation (NCT01029353), and evaluations of surgical devices like ventriculoperitoneal shunts in neonates with post-hemorrhagic hydrocephalus, where NEC may be a complication (NCT00115934) [34].

#### 3.2.7. Medical Devices

Medical devices were evaluated in 3 studies, with NEC as a secondary outcome in 2 neonatal studies. Only one of these studies was completed, with reported results [35]. Devices studied included implementable medical devices capable of lengthening intestinal tissue for patients with short bowel syndrome (NCT05535361), cycled light exposure during phototherapy (NCT03927833), as well as maternal interventions like the use of a cervical pessary to reduce the incidence of preterm birth (NCT02901626).

#### 3.2.8. In Vitro Approaches

In vitro studies consisted of 2 trials, both of which designated NEC as the primary outcome. These studies utilized tissue samples from preterm infants to investigate the role of neutrophil extracellular traps (NETs) in the pathogenesis of NEC. Only one of the studies had reported results, though the study itself was terminated (NCT01106209). The focus was on identifying inflammatory pathways that contribute to the development of NEC in the neonatal immune system.

Taken together, these ongoing and completed studies reflect the direction of NEC research: prevention, medical management, optimization of surgical treatment, and diagnosis. There is a distinct focus on preventing preterm delivery through maternal intervention by a variety of approaches, and there are neonate-focused approaches to improve respiratory function and vascularization, key risk areas in the pathogenesis of NEC. Regarding clinical management (both medical and surgical) of NEC, the variety and diversity of modalities currently under investigation reflect the multifaceted nature of NEC. It also highlights the need for further treatment as well as basic science studies to understand NEC pathogenesis.

## 4. Diagnostic Biomarkers

The current diagnosis of NEC is based on a combination of clinical, laboratory, and radiologic findings. There are several well-established markers, including procalcitonin (PCT), serum amyloid-A (SAA), platelet-activating factor, hepcidin, toll-like receptors, cytokines (IL-6, IL-8, TNFα), and chemokines. However, they are common inflammation-driven molecules that are not specific to NEC, and thus, may not fully allow for differentiation between NEC and other inflammatory diseases [36,37,38]. Furthermore, their diagnostic value is often limited due to fluctuating levels before, during, and after NEC development, and it depends on disease severity. Over the last decade, numerous studies have investigated plasma, urine, and fecal levels of various markers to identify biomarkers that can predict disease onset, pathogenesis, and future complications. These investigations have been significantly enhanced by the use of advanced techniques, including OMICS experiments and artificial intelligence (AI) [39,40,41].

### 4.1. Inflammatory Biomarkers

Due to the inherent inflammation-driven biology of NEC, there are inflammatory biomarkers associated with the early-, mid-, and late-phases of NEC. As such, IL-6 and IL-8 levels surge within 1–2 h of infection, PCT between 3 and 4 h with levels detectable up to 24 h, neutrophil CD64 rising at 1–6 h and persisting at 24–48 h, and C-reactive protein (CRP) showing the highest levels at 1 and 2 days of infection. A study on 192 preterm infants with several inflammatory characteristics, including sepsis, NEC, and bronchopulmonary dysplasia, followed for one year, showed that serial analysis of CRP and PCT is a promising biomarker for identifying infants at risk of growth failure [42].

The potential of combining these markers to enhance decision-making for therapeutic approaches is a significant advancement in NEC management. This approach not only boosts the sensitivity and specificity of the diagnosis but also provides a practical tool for clinicians. The increased levels of IL-6 and IL-8 lead to the synthesis of SAA in various cells, which, when combined with the assessment of apolipoprotein CII, provide an opportunity to identify infants with NEC at the time of diagnosis. Another marker, soluble triggering receptor expressed on myeloid cells-1 (sTREM-1), with a specificity and sensitivity range of 70–100%, has been suggested as a late-phase marker predicting septic shock and death [43]. The inclusion of advanced whole blood cell indices, such as mean neutrophil volume, mean monocyte volume, conductivity, scattering, and distribution width, has been considered. However, their lower specificity and sensitivity compared to CRP and PCT, due to the significant fluctuation in white blood cell counts during disease development, mean they offer little to no improvement.

Regulatory molecules have also been considered as potential biomarkers. For instance, one study investigated whether Resistin and Resistin-like molecule b (RELMβ) can be utilized as biomarkers. Results showed that while Resistin levels do not allow differentiation between NEC (Bell’s stage II) and controls (preterm infants), the sensitivity and specificity of RELMβ concentrations in the plasma of neonatal NEC were 71.4% and 91.7%, respectively, and were significantly higher in NEC patients compared to controls. This was significantly improved by the combination with thrombocytopenia, which resulted in 82.89% sensitivity and 93.21% specificity, respectively [44].

The imbalance between Treg and Th17 levels is a characteristic trait of NEC inflammation. A study employing patients’ data and a mouse animal model showed that CCR9^+^CD4^+^T cells in peripheral blood are significantly increased in NEC conditions, making this specific cell subtype a potential severity biomarker [45].

As mentioned before, there are biomarkers in blood that allow disease diagnosis; however, blood collection in these infants can lead to anemia and worsen the outcome. Additional drawbacks include waiting time for bacterial culture or the lack of sensitivity of the assays. Thus, methods that allow for the detection of bacterial infections and inflammatory markers in saliva would be beneficial for disease diagnosis and follow-up. In a limited study of forty-five babies with 16 bacterial flares in 10 preterm-born neonates, IL-6, IL-8, macrophage inflammatory protein (MIP)1 α, MIP-1β, and tumor necrosis factor (TNF) α were also detected alongside significantly higher levels of blood CRP and sugar during the infections [46]. Interestingly, a combination of increased IL-6 and sugar levels accurately detected bacterial infections. The potential of saliva testing to revolutionize NEC diagnosis is a hopeful prospect that could significantly improve the diagnostic process.

Another study [47] collected plasma samples from premature infants with NEC (n = 30), sepsis (n = 29), and controls (n = 29) and assessed the levels of 92 inflammation-related proteins. The authors identified eleven factors, including IL-8, TRAIL, IL-24, MMP-10, CCL20, CXCL1, OPG, TSLP, MCP-4, TNFSF14, and LIF, that significantly differed among NEC, sepsis, and control group. They demonstrated that using a combination of them could increase the sensitivity and specificity of the NEC detection. As such, they showed that the combination of IL-8, OPG, MCP-4, IL-24, LIF, and CCL20 could distinguish between stages II and III of NEC. At the same time, combining IL-8, IL-24, and CCL20 provided the best predictive value for comparisons between NEC and sepsis or control, enabling the detection of differences in NEC severity.

Sullivan and colleagues evaluated eleven inflammatory biomarkers and a pulse oximetry sepsis warning score (POWS) to determine whether their combination allows for early diagnosis of late-onset sepsis (LOS) and NEC [48]. Their study encompassed 188 samples from 54 very low birth weight infants with analysis of seven biomarkers (IP-10, IL-6, IL-10, IL-18, TNFα, IL-8, PCT) during the entire study, while assessment of four HGF, EGF, sST-2, and IL1ra was successfully measured 80% of the time. In addition, they assessed the levels of bacteremia, and of the 28 cases of LOS or NEC with bacteremia, 22 were Gram-positive (GP) bacteremia, 5 Gram-negative (GN) bacteremia, and 1 had NEC with GN bacteremia. IL-6, TNFα, IL-8, IL-10, and sST2 were shown to be increased in infants with GN sepsis or NEC compared to those without these conditions. Furthermore, the levels of IL-6, with 78% specificity at 100% sensitivity, allowed for the detection of GN LOS or NEC. A combination of inflammatory biomarkers obtained from plasma could potentially discriminate sepsis due to GN bacteremia or NEC.

As inflammatory processes at the fetal-maternal interface cause more than half of preterm births, Zaharie and colleagues evaluated whether combining maternal inflammation with data from infants can improve NEC diagnosis [49]. The study included 82 preterm babies, from whom 20 progressed to NEC, and evaluated maternal inflammatory status such as CRP, chorioamnionitis, preeclampsia, and several neonatal inflammatory markers, including CRP 1, CRP 2, PCT, IL-3, and MMP9. The results showed that neonates who progressed to NEC had higher levels of IL-3 at birth, and there was a significant positive correlation with maternal CRP levels, suggesting that these markers could potentially be used as predictive indicators for NEC.

Gasdermin D (GSDMD), another inflammatory marker that activates programmed cell death and release of several pro-inflammatory cytokines, was assessed alongside IL-1β in the study, which included 59 stage II NEC patients and 56 matched controls [50]. The laboratory results showed that patients with NEC had lower neutrophil and red blood cell counts, higher platelet counts, and increased levels of CRP and PCT. This was accompanied by significantly increased levels of GSDMD and IL-1β, a novel finding that positions them as early biomarkers for NEC prediction.

### 4.2. Fecal Biomarkers

One of the best characterized fecal biomarkers for the early detection of NEC is Calprotectin (CP) [51,52]. CP is expressed in innate immune cells, specifically by neutrophils, activated monocytes, and macrophages [53]. The fecal CP levels are directly correlated to the levels of leukocyte recruitment to the inflamed small intestinal tissue. Therefore, elevated fecal CP concentrations are seen in infectious and inflammatory gastrointestinal diseases such as NEC.

Initially, CP, due to a high degree of stability, was thought to be a promising biomarker for NEC. However, a systematic review of thirteen studies, including 601 NEC patients, showed that the sensitivity of detecting NEC using CP as a marker ranged from 76% to 100%. In contrast, specificity ranged from 39 to 96.4%, with varying cut-off values [54]. Thus, CP holds some predictive value, but it is not a perfect biomarker. The limitations of CP as a biomarker, including its varying sensitivity and specificity, suggest the need for further research to identify more reliable biomarkers or to develop a panel of biomarkers for NEC diagnosis. Some studies combine CP levels with other inflammatory markers. One such study, with 18 controls, 7 suspected NEC patients (Bell’s stage I), and 5 NEC-diagnosed infants (Bell’s stage II), investigated the correlation between fecal CP and gut inflammation in neonates with congenital heart defects. Their study showed that patients with NEC stages I and II had significantly increased levels of fecal CP [55]. In two of the NEC stage II patients, the fecal levels of CP were increased before the diagnosis and radiological assessment. In another study [56], which included 8 infants with stage III NEC and 14 non-NEC controls, the fecal levels of CP and lipocalin-2 were sufficient to predict NEC one week before diagnosis. Adding one or two other markers, such as prostaglandin E2, lysozyme, alkaline phosphatase, or haptoglobin, did not improve the diagnosis, as controls were falsely identified.

Another candidate NEC biomarker, S100A12, was considered because inflamed intestinal mucosa releases S100A12. For example, a study has shown significantly higher levels of fecal S100A12 in infants with stage III NEC at the onset of the disease [57]. In contrast to fecal CP, the levels of fecal S100A12 were significantly increased 4–10 days before the onset of NEC, suggesting its potential as an early biomarker/predictor. Another study evaluated four proteins, Human β-defensin 2 (HBD-2), Claudin-3, high-mobility Group box-1 protein (HMGB-1), and RELMβ, and their ability to predict NEC severity [58]. All these markers have been previously investigated for their suitability as biomarkers for NEC, as they are all dysregulated in response to intestinal inflammation. Liu et al. [58] studied 27 infants diagnosed with NEC stage III and 33 infants diagnosed with NEC stage II. Bell’s stage III patients had fecal samples with significantly elevated levels of HBD-2 and Claudin-3, and a decreased neutrophil count compared to patients with Bell’s stage II. However, neither the levels of RELMβ nor HMGB-1 could distinguish between these two stages.

The impact of NEC on gut physiological function has led to the exploration of novel approaches to NEC diagnosis, such as fecal gas analysis. The studies by the de Boer group [59] analyzing 13 NEC patients and 14 controls show that fecal Volatile Organic Compounds (FVOCs) can be used to predict NEC within one to two days of symptom onset. In another study, with 32 NEC patients and 70 controls, FVOCs were found to cluster into nine groups. Three of them were associated with NEC and indicated the possibility of identifying the disease 3–4 days before the clinical diagnosis [60]. While these findings are promising, the current literature is limited. However, recent studies combining FVOCs and microbiota analysis have identified 8 unique FVOC features. Unfortunately, as the authors stated in their paper, the data are not consistent with previous findings, which impeded the validation and use of FVOC in clinical practice [59,60,61].

### 4.3. Metabolomics and Metagenomics-Based Markers

There is a consensus that changes in the microbiota precede the development of NEC, thereby allowing for the identification of microbes correlated with disease development. More specifically, intestinal microbial communities of healthy breast-fed infants are dominated by bifidobacterial species, mainly *Bifidobacterium bifidum* and *Bifidobacterium longum* subsp. *infantis*, while NEC patients had an increased abundance of *Clostridium* and *Enterobacteriaceae* genera [62,63,64,65].

Metabolomics and metagenomics assessments of 60 urine and stool samples revealed colonization by *Firmicutes* or *Proteobacteria*, which preceded the development of NEC, while *Propionibacterium* was found only in control samples. Additional correlation between urine alanine was significantly higher in NEC preceded by *Firmicutes*, while histidine was considerably lower in NEC preceded by *Proteobacteria* dysbiosis [66]. This study showed more of the association between the bacteria and disease development.

A limited study of urine samples from 6 infants who developed NEC and 12 age-matched controls demonstrated constant changes in the metabolome in all recruited infants, except in cases of NEC occurring after the 40th day of life. In NEC cases, increased urinary gluconic acid levels were found, which were attributed either to the gluconic calcium administration or to a byproduct of glucose oxidation or microbiome metabolism [67].

In a case–control study examining changes in the serum proteome and metabolome conducted by Stewart and colleagues, eight proteins were associated with NEC, and four proteins with LOS. This study is significant as it confirmed previously identified markers of NEC, such as CRP, Ig a-2 chain C region, and macrophage migration inhibitory factor, but failed to identify any new markers [68]. Metabolomics revealed that specific metabolites involved in C21-steroid hormone biosynthesis, linoleic acid metabolism, and arachidonic acid pathways were associated with NEC development [68]. The study supported a protective effect of gut microbiome diversity, characterized by a high abundance of *bifidobacteria*, on NEC development [69]. However, the authors demonstrate that NEC patients do not have a unique microbial signature. Interestingly, unlike the study mentioned above, Victor and colleagues were unable to identify any differences in metabolomics that would enable the discovery of biomarkers capable of reliably detecting infants at high risk for developing NEC [70]. Another study demonstrated that fecal metabolomes are unique to each individual and change over time, indicating that they are not associated with NEC or other health outcomes [71].

A study showing that sphingomyelins are increased and ceramides are decreased in Bell’s stages II and III of NEC patients provides a rationale for evaluating them as potential biomarkers of NEC. One of the first studies demonstrated that there are similar levels of these markers between Bell’s stage I and controls, which may help predict NEC development [72]. However, as the authors stated, the significant variability in the control samples prevented achieving the necessary accuracy to develop a machine learning model for NEC prediction.

A pilot study evaluating 15 neonates with NEC and 15 matched controls demonstrated a difference in metabolic profile between neonates with NEC and the controls. Further analysis revealed that levels of urine tyrosine, arginine, and riboflavin can serve as surrogate biomarkers for NEC diagnosis [73]. The survey by Sylvester and colleagues confirmed metabolic differences at birth in premature-born children. These changes are progressing before NEC development, and significantly utilize dried blood spots, providing advantages compared to other methods [74].

The advancement of high-throughput methods enabling direct whole-genome and 16S RNA sequencing of stool samples presents a promising avenue for utilizing changes in the microbiome of NEC patients as biomarkers. However, the data indicate a limited diversity of the microbiota in infants, with only four class-level taxa. Studies demonstrate significant variability in the effect of NEC on microbiota, with some studies pinpointing changes and others showing no significant impact. Longitudinal studies show changes in some microbial compositions, such as an increase in Gram-negative bacteria and a decrease in anaerobes (classes *Clostridia* and *Negativicutes*). A significant limitation of most of these studies is the extremely low number of NEC cases and the lack of consistency in timepoint collections, sample source, and analysis techniques.

A meta-analysis of 47 shotgun sequencing datasets revealed that NEC subjects exhibit an increase in opportunistic microbial species, which is accompanied by a loss of gut microbial biodiversity [75]. Moreover, this study showed that *Clostridium eonatale* and *perfringens* appeared before NEC development and suggested DL-lactate as a putative metabolic biomarker for early detection of NEC onset.

A pilot study comparing gestational ages in the NEC group (n = 17) and the non-NEC group (n = 17) concluded that there was an increase in *Streptococcus salivarius* and *Rothia mucilaginosa*, and a decrease in *Bifidobacterium animalis* subsp. *lactis*, and the reduction of acetic, propionic, and butyric acids, could be used to diagnose early NEC [76]. Another study evaluated LOS and NEC to identify metabolic markers that can differentiate between these two states [77]. Analysis of blood samples from 15 septic neonates and 17 neonates with NEC at clinical suspicion revealed significant differences in the metabolic profiles compared to controls. Specifically, phosphatidylcholines or lysophosphatidylcholines were found to be significantly reduced both in neonates with LOS and NEC compared to controls. Furthermore, the authors showed that L-carnitine could distinguish NEC cases from controls.

Baraldi et al. conducted a systematic review of studies that apply untargeted metabolomics and gut microbiota analysis to identify predictive markers for NEC [78]. The study found that at the phylum level, NEC samples were characterized by increased relative abundance of Proteobacteria compared to controls. At the genus level, pre-NEC samples were characterized by a lack or decreased abundance of *Bifidobacterium*. Finally, at the species level, *Bacteroides dorei*, *Clostridium perfringens*, and perfringens-like strains dominated early NEC specimens, whereas *Clostridium butyricum*, *neonatale*, and *Propionibacterium acnei* characterized the disease diagnosis. In contrast, 16S rRNA gene amplicon sequencing shows only minor differences in microbiota profiles at the early stages of NEC [79]. At the same time, there are more significant changes at the Bell stage II and III, characterized by a decrease in the abundance of *Enterococcus*. These were accompanied by an increase in fecal cytokine levels (IL-1α, IL-5, IL-10) compared to controls.

A recent prospective study on infants born before 34 gestational weeks, with longitudinally collected urine analysis, included 35 patients with NEC, 5 with spontaneous intestinal perforation, 14 patients with other GI diseases, and 113 controls. The study employed untargeted metabolomic analysis based on mass spectrometry [80]. The authors demonstrated that surgical NEC patients had higher N-acetylaspartic acid, butyrylcarnitine, and propionylcarnitine levels than those with medical NEC. The early urinary metabolome has the potential to predict surgical necrotizing enterocolitis (surgical NEC), which represents a significant advancement in NEC research.

### 4.4. Proteomics Biomarkers

A comprehensive review of biomarkers based on metabolomics and proteomics can be found elsewhere [81]. Here, we mention the most recent approaches to biomarker identification. For example, a study employed LC-MS/MS on blood samples from NEC, LOS, and the control group, suggesting two panels with three proteins each with high and significant value in diagnosing LOS (CRP, CETP, and APOA4) and differentiating between LOS-NEC transformation (APOA4, APOC1, and LCAT) [82].

Studies have also shown that human milk oligosaccharides (HMOs) present in a mother’s breast milk are protective against NEC. Unfortunately, there are some cases of breast-fed infants who developed NEC, which can be a result of the composition of the mother’s milk. Thus, studies investigating whether the correlation between HMO and infant microbiome may predict NEC are of interest. Studies from a neonatal rat model demonstrated that disialyllacto-N-tetraose (DSLNT), a non-fucosylated but double-sialylated HMO, significantly reduces NEC development and improves NEC-associated mortality rate [83]. The potential of DSLNT as a predictive biomarker for NEC diagnosis is a promising avenue for future research, offering hope for improved diagnosis and treatment. Instability of the gut microbiome in infants with NEC has also been reported in longitudinal studies, with more frequent transitions between different preterm gut community types in NEC. However, bacteria such as *Bifidobacterium* spp. have been established as being relevant to infant well-being. The studies were conducted on 33 patients with NEC and 37 controls, with 19 HMOs assessed. Out of 19 HMOs, only DSLNT was able to predict NEC diagnosis. However, as the levels of DSLNT identified 100% NEC cases, it additionally identifies controls at 60%. The metagenomic analysis confirmed that before the onset of NEC, infants have decreased levels of *Bifidobacterium* spp., specifically *B. longum*, and an increased abundance of *E. cloacae* [83].

Another study, in a cohort of 12 infants diagnosed with NEC and 6 matching control infants, utilized an aptamer-based proteomic discovery assay to identify serum biomarkers. The authors compared levels of serum proteins in neonates with and without NEC and identified ten differentially expressed serum proteins between these groups. Out of 1300 serum proteins analyzed, 10 proteins, including 2 increased (C-C motif chemokine ligand 16 and immunoglobulin heavy constant alpha 1 and 2) and 8 decreased (collectin subfamily member 12, glucagon, alpha fetoprotein, formimidoyltransferase cyclodeaminase, matrix metallopeptidase 13, glycoprotein hormone alpha polypeptide heterodimer, MHC class I polypeptide-related sequence A, and Ephrin A3) in relative abundance were significantly different in NEC compared to the controls [84]. All these markers showed high sensitivity versus specificity levels for NEC detection. These findings open up new possibilities for further research and may lead to the development of a potential diagnostic tool.

Another group employed a combination of immunofluorescence methods and enzyme-linked immunosorbent assay (ELISA) to identify proteins differentially expressed in the intestinal tissues of NEC patients, comparing the necrotic and normal segments [85]. The technique allowed for the identification and quantification of 6880 proteins. Out of these, 55 were significantly upregulated and 40 were downregulated in the necrotic sections compared to the normal sections. The identified proteins are involved in biological processes such as mitochondrial organization, vasoconstriction, rRNA catabolism, fluid shear stress response, and glycerol ether biosynthesis. Secondary analysis showed that tumor necrosis factor receptor-associated factor 6 and IL-8 were significantly upregulated in the intestinal tissues and serum samples of patients with NEC.

### 4.5. Genomic and Transcriptomics-Based Markers

#### 4.5.1. Genomic-Based Markers

In recent years, the search for biomarkers for NEC has been expanded due to advancements in the fields of genomics and transcriptomics. Studies have shown that silencing of genes by hypermethylation (inactivation) of their promoters, changes to the expression levels of genes, or a substitution of a single nucleotide leading to single-nucleotide polymorphisms (SNPs), can result in deregulation of gene expression or abnormal activity of the gene product. The identification of widespread regions of hypermethylation in samples from the small intestine, colon, peripheral blood, and stool compared to controls offers a new category of biomarkers for NEC diagnosis [86,87,88,89,90]. The ongoing investigation into SNPs in inflammation-associated genes may help elucidate the correlation between minor alterations in the nucleotide sequences of coding and regulatory elements and genes implicated in NEC development and progression [91].

In the cohort of 184 premature infants, with 118 controls, 22 NEC stage II, and 44 NEC stage III diagnosed infants, SNP analysis of Tripartite motif containing-21 (TRIM21) gene, a receptor for antigen–antibody complexes, and inflammatory cytokines (IL-1β, IL-6, IL-12, NOS3, PXR, TGFβ1, TLR4, TNFα) was performed. The authors found that the TGFβ1 SNP, rs2241712, resulted in a reduction in NEC-associated intestinal perforation but an increase in mortality. At the same time, the TRIM21 SNP, rs660, was also associated with increased incidence of perforation, and the IL-6 SNP (rs1800795) with increased incidence and severity of NEC [91].

Research by Sampath et al. [92] has shed light on the protective role of genetic variants in NEC. Their evaluation of the key autophagy gene, ATG16L1, revealed that a common ATG16L1 variant, where the threonine at position 300 is substituted by alanine (rs2241880, Thr300Ala), confers protection against NEC. This finding, supported by an independent cohort (260 infants, 23 with NEC), suggests a potential avenue for preventive measures, demonstrating that decreased autophagy due to loss-of-function variants in autophagy genes showed a trend towards decreased NEC [92].

The vascular endothelial growth factor (VEGF), a heparin-binding gene critical in angiogenesis, has been studied as a potential susceptibility factor for NEC. It has been demonstrated that an SNP (VEGF-2578) leads to reduced VEGF levels, thereby increasing susceptibility to NEC [93]. On the other hand, a more recent study was unable to confirm these early observations [94], and the authors cited possible limitations, including the inclusion criteria and ethnic background of the patients. Meanwhile, two other variants of VEGFA (rs699947 and rs833061) have been found to increase the risk of NEC when studied in the Chinese Han population [95].

A susceptibility to developing NEC is caused by genetic differences in genes that modulate the immune and inflammatory response. Dual Specificity Phosphatase 1 (DUSP1) deficiency increased pro-inflammatory cytokine production in intestinal epithelial cells, as well as enhanced inflammation-induced apoptosis, leading to epithelial barrier dysfunction and subsequent inflammation and cell death. In a small pilot study (50 cases and 38 controls), analysis of 31 inherited SNPs in DUSP genes showed that the presence of the rs704074 SNP in the DUSP6 gene was associated with a 48% decreased risk of developing NEC and a 78% reduced risk of surgical NEC for each copy of the rs704074/G [96].

Another study combined single-cell RNA-sequencing data with DNA methylation status to identify a set of methylation-related differentially expressed genes (MrDEGs). Thus, genes with an increased methylation status in their promoter regions should have their corresponding RNA transcription levels decreased [97]. In the cohort of eight infants with NEC and matched controls, this study confirmed a higher level of hypermethylation in NEC, predominantly in the colon, with 994 and 262 increased methylation regions in the ileum. These MrDEGs included genes such as ArfGAP with dual PH Domains 1 (ADAP1), Guanylate Cyclase Activator 2A (GUCA2A), BCL2 Like 14, Fucosyltransferase 3, Mitotic Spindle Positioning (MISP), Usher syndrome type 1C, ITGA3, Unc-93 Homolog A, and IL-22RA1. Confirmatory studies verified that the methylation status of their promoters regulates the expression of the ADAP1, GUCA2A, IL-22RA1, and MISP genes, and that their expression levels were significantly reduced in NEC compared to controls. In contrast, the others did not reach significance or showed differential methylation between the ileum and colon.

#### 4.5.2. Transcriptomics-Based Markers

One of the highest contributors to preterm birth is an increased inflammation of the fetal membranes and amniotic fluid within the uterus (chorioamnionitis), accounting for 40–70% of cases and causing fetal inflammatory response syndrome [98,99,100]. As such, infection before birth in neonates results in higher levels of the Th1 response, which is predominantly responsible for defense during infection. The comparison of the transcriptome in whole blood samples from neonates exposed to chorioamnionitis and uninfected controls revealed a clear difference in gene expression patterns. Specifically, the authors identified 488 genes that were significantly differentially expressed, most of which belonged to the innate and adaptive immune response pathways. The most differentiated genes were components of active pathways, such as Ras-related C3 botulinum toxin substrate (Rac) signaling, N-formyl-methionyl-leucyl-phenylalanine (fMLP) signaling in neutrophils, FC receptor-mediated phagocytosis in macrophages and monocytes, FC epsilon receptor I (FCεRI) signaling, and G beta-gamma (Gβγ). In-depth analysis utilizing multiple computational tools enabled the authors to identify *microRNA-155* as a major regulator of identified pathways [101]. Notably, *miR-155* is upregulated during inflammation to stimulate Th1 and Th17 inflammatory responses [102].

In recent years, microRNA (miR) and miR-regulated networks have emerged as promising biomarkers in inflammatory diseases [103]. MiRs are short, single-stranded, non-coding RNA molecules that regulate gene expression, protein translation, and cell-to-cell communication [104,105]. These networks play a crucial role in NEC pathophysiology [106]. The combined analysis of differentially expressed miRNAs and their targeted genes has identified *miR-429/200a/b* and *miR-141/200c* clusters that are expressed at low levels and potentially play a significant role in NEC. Simultaneously, the genes regulated by these miRs, such as VEGFA, E-selectin, kinase insert domain receptor, fms-related tyrosine kinase 1, and hepatocyte growth factor, showed increased expression in NEC [107]. Besner and colleagues identified several miRs with levels significantly altered in infants with NEC compared to controls, including *miR-376a*, *miR-518a-3p*, and *miR-604* [108]. The role of these miRs in NEC has not been fully understood. However, studies from other inflammatory diseases suggest that further mechanistic investigations of these miRs are warranted [109,110]. Importantly, this non-invasive, urine-based approach to NEC diagnosis offers a ray of hope, potentially revolutionizing the way we detect and treat this condition.

### 4.6. Others

Among the nine fatty acid-binding proteins (FABPs), three are linked to the gastrointestinal tract, with FABP2 emerging as a promising biomarker in NEC. FABP2 is highly expressed in the intestinal epithelium and plays a crucial role in lipid metabolism, influencing proliferation, barrier function, and inflammation [111]. The potential of FABP2 as a biomarker for NEC is promising, with increased levels of circulating FABP2 being associated with increased risk of subsequent NEC. A recent meta-analysis, based on data from 262 infants with NEC and 310 healthy controls, confirmed a positive association between serum or urine FABP2 levels and the presence of NEC. This study also revealed an increase in FABP2 levels in advanced-stage disease (Bell’s stage ≥ 2) compared to early-stage disease. However, two studies show only moderate sensitivity but high specificity of FABP2, indicating the need for further research to understand its full potential. It is also unclear whether FABP2 levels, either alone or in combination with additional markers, can be used to guide decisions regarding surgery [112]. A comparison of samples from 30 human neonates with NEC and 30 healthy neonates revealed that serum levels of FABP2 mRNA and protein, as well as IL-6 levels, were significantly increased in NEC cases [113]. Additionally, they showed that a combination of these markers can have predictive value.

Given the essential role of Paneth cells in responding to inflammatory insults, the proteins they produce could potentially serve as markers for NEC. Markasz and colleagues used semi-automated digital image analysis of immunohistochemical tissues to investigate whether the levels of expression of defensin alpha 6 (DEFA6) and GUCA2A differ between NEC and control groups [114]. The study, which included 43 NEC cases and 27 controls, found that DEFA6 expression was lower in the NEC group, correlating with the risk of developing NEC, independently of gestational age and birth weight, while GUCA2A levels did not differ.

Given the observed differences in hematological features and blood parameters between controls and NEC patients, these could be considered as markers to inform the diagnosis. For instance, lower platelet counts were observed at the onset of NEC in surgical cases of NEC patients, specifically in those with a gestational age below 28 weeks [115]. In patients between the gestational ages of 28 and 32 weeks, the analysis showed decreased levels of lymphocytes, as well as reduced percentages of platelets, lymphocytes, and monocytes, while between weeks 32 and 37, only the lower absolute counts and percentages of lymphocytes were noted. Similarly, a retrospective study of 79 patients with mild-to-moderate NEC and 43 patients with severe NEC investigated whether intestinal tissue oxygen saturation (r_int_SO2) can serve as a predictive marker for post-surgery survival in patients with NEC. Besides the r_int_SO2, the study evaluated the white blood cell count, platelet count, PCT, MPV, red blood cell distribution width, hemoglobin, and CRP between these two groups. The analysis showed that rintSO2 was reduced, while PCT and MPV were increased in the severe NEC cases compared to the mild group. A combination of three factors: r_int_SO2, PCT, and MPV was suggested as a good predictor of NEC severity.

In summary, despite progress made toward identifying biomarkers for NEC, we still lack a reliable marker(s) that can be easily employed to predict NEC development and disease progression. Various factors, including sample size, methodological variabilities, testing times, and data reporting, hamper the search.

## 5. Experimental Models of Necrotizing Enterocolitis

In order to uncover potential novel therapies for necrotizing enterocolitis, basic and translational science models are crucial. These models allow researchers to study molecular mechanisms of the disease to identify therapeutic targets, and subsequently test them in a preclinical setting. In vivo animal models of NEC have been foundational to expanding our understanding of NEC pathogenesis; however, the development of more advanced in vitro methods has also enabled in vitro modeling of NEC. In this section, we discuss notable in vivo and in vitro models of NEC, with an emphasis on those that have been applied within the last decade.

### 5.1. In Vivo Models of NEC

Animal models of NEC date back to the early 1970s [116], about a decade after the establishment of the first NICU in the United States. The earliest NEC animal models were attempted in many animal species, including pigs, rodents, rabbits, guinea pigs, and goats [116]. Since then, in vivo NEC models have focused specifically on rodents and pigs. Using animals to study NEC allows for modeling within a complex whole-body system and is essential for our understanding of the disease [117].

#### 5.1.1. Rodents

Mice and rats are widely used in translational science research to study human diseases, and this is also the case for the field of NEC research. The earliest rodent NEC models using rodents applied cold stress or hypoxia in formula-fed newborn rats [118]. Barlow and Santulli (1975) predicted that hypothermia would decrease mesenteric blood flow and induce bowel ischemia [118]. They also concluded that a higher number of hypothermic or hypoxic exposures would result in worse disease pathology [118]. Combined hypoxia and hypothermia, along with formula feeding, are still used to model NEC in both C57BL/6J [119] and BALB/c mice [120].

**Figure 3:**
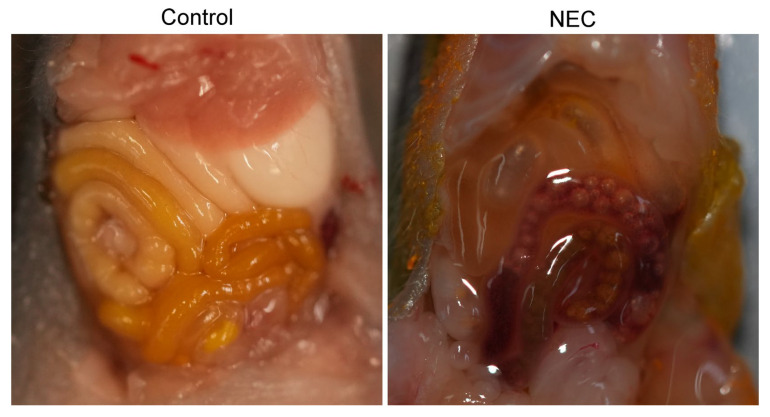
**A mouse model of NEC using dextran sodium sulfate.** Healthy breastfed mice (left) contain intact intestines full of breastmilk. In comparison, formula feeding with DSS induces NEC pathology in neonatal mice [112], with evident abdominal distension, bloody stool, intestinal gas, and bowel damage. Images were taken at Stony Brook University by Cuilee Sha (author).

Formula feeding in human neonates is an important risk factor for NEC; however, not all formula-fed infants will develop NEC, so other factors need to be considered. Dextran sodium sulfate (DSS) is an osmotic agent that induces inflammatory bowel disease in adult animals. For this reason, it was theorized that administration of DSS in neonatal rodent animals could induce an intestinal pathology that mimicked NEC. In both breastfed neonatal rat pups that received DSS in their water and formula-fed neonatal mouse pups that received DSS in the formula [121,122], animals displayed intestinal pathology that was characteristic of NEC (Figure 3). Additionally, it was found that changing the concentration of DSS added to the formula and administered to neonatal mice correlated with the severity of the disease induced [122].

It is also well-established that the composition of the gut microbiome is altered in NEC patients compared to age-matched premature neonates [123]. Therefore, to mimic a change in the gut microbial environment, rodent models of NEC have supplemented formula feeding with additional gastrointestinal insults. To parallel the gut microbiome in NEC, certain research groups have isolated enteric bacteria from the bowels of NEC patients and added them to formula [124,125]. A potential issue with this method is that various environmental factors influence the gut microbiome and may vary depending on the patient or hospital from which it was derived, making reproducibility challenging across different groups from different locations. To avoid this confounding variable, researchers instead looked at bacterial inflammatory output, specifically from Gram-negative bacteria that are abundant in early life [123]. They discovered lipopolysaccharide (LPS) [126], which can activate the immune system via the TLR4 pathway; with the understanding that systemic immune activation is a well-known complication of NEC.

Finally, another critical attribute of NEC is that it is a disease of prematurity. Therefore, in rat models specifically, certain studies have performed cesarean section on pregnant dams at around 20 days of gestational age to deliver premature rats [127]. After delivery, the pups are raised on formula with hypoxic and hypothermic stressors as discussed previously.

#### 5.1.2. Pigs

Even before rodent models, neonatal piglets were one of the first animals used to model NEC [116]. These early groups would induce NEC by exposing full-term piglets to asphyxiation [128]. Fortunately, these practices are no longer implemented, though these studies established important foundational work for the development of subsequent animal models of NEC. More current studies on NEC using piglets have instead focused on isolating premature piglets [129,130], and then supplementing their growth with formula feeding and ischemia [131], similar to the rodent models. Although pigs, in general, have a gastrointestinal system that more closely resembles that of humans and are more similar in size, the cost and effort of maintaining such large animals have made rodents the primary animal models of NEC. Table 3 summarizes the current major animal models of NEC, including specifics on the NEC induction protocols.

### 5.2. In Vitro Models of NEC

Although animal models are still widely used to study NEC and are crucial for understanding systemic effects and organ-organ interactions, in vitro models have an essential application in translational NEC research, enabling the identification and isolation of specific molecular pathways. As in vitro models become more complex, these studies are better at learning about broader systemic effects. Additionally, using in vitro models allows for sampling from human cell lines and human tissue, thereby providing a better understanding of the disease as it affects human neonates.

#### 5.2.1. Cell Lines

The rat intestinal epithelial cell line-6 (IEC-6) is a cell line of homogeneous epithelial cells. To study diseases using IEC-6, these cells need to be exposed to external insults. For NEC specifically, this insult is LPS [132], as used in animal models, with or without cobalt chloride [133]. LPS alone resulted in decreased proliferation, increased apoptosis, and autophagy in the IEC-6 cells [132]. The combination of LPS with cobalt chloride further showed a decrease in cell viability, an increase in systemic inflammatory markers like IL-6 and TNFα, and a decrease in tight junction proteins. These findings suggest that the LPS-cobalt chloride combination results in severe inflammation and barrier dysfunction that are characteristic of NEC [132].

Human intestinal cell lines that have been used to study NEC include the HT-29 and Caco-2 cell lines [134]. Although the Caco-2 cell line appears more frequently in the literature, both cell lines are derived from patients with colorectal adenocarcinoma. NEC induction in the HT-29 cell line was done through bacterial administration, specifically with *E. coli* or *C. sakazakii* [134,135]. In the same study that exposed HT-29 cells to *C. sakazakii*, Caco-2 cells were also exposed to this bacterium and found to have increased intestinal inflammation and decreased barrier stability [134]. Another group that used the Caco-2 cell line to study NEC induced the disease through LPS inoculation [136,137]. They found that LPS application to Caco-2 cells led to increased intestinal permeability and the expression of tight junction proteins such as claudins, and that inhibition of Rho kinase protects against these effects [136,137]. Finally, a 2021 study suggested co-culturing Caco-2/HT29-MTX cells to investigate intestinal properties [138]; however, this model has not been applied explicitly to NEC, but there is potential for its use.

#### 5.2.2. Organoids and Enteroids

Much of in vitro research has advanced from using cell lines to generating organoid models, which have a complex three-dimensional structure and can be derived directly from subjects with the disease of interest. This shift has also been evident in NEC research, as the latest literature in this field has leveraged the use of organoids. A 2024 study used mouse organoids generated from postnatal day 9 (P9) mice that had NEC induced between P5–P9 using formula with LPS, hypoxia, and hypothermia [139]. This study found that treatment with human milk oligosaccharides protected against intestinal injury and promoted cell proliferation and stem cell activity [139].

Similarly, in humans, organoids have been successfully cultured from premature neonates with NEC, allowing for the study of the disease in the laboratory [140,141]. The use of organoids to study NEC was confirmed by increased inflammation, specifically in the expression levels of TNFα and IL-8 [141], increased apoptosis, and decreased expression of tight junction proteins upon LPS challenge [140]. Interestingly, organoids derived from patients who had recovered from NEC also demonstrated an increase in inflammatory marker expression upon LPS challenge, but not at baseline compared to control organoids from patients without NEC [141]. To improve upon the organoid model, another group developed a microfluidic model of NEC, called “NEC-on-a-chip”, which co-cultures intestinal enteroids derived from a preterm neonate without an inflammatory intestinal condition with human endothelial cells and a microbiome derived from an infant with severe NEC [142]. Compared to intestinal enteroids cultured without the dysbiotic NEC microbiome, the “NEC-on-a-chip” model saw an increase in pro-inflammatory cytokines IL-1β and IL-8, decrease in proliferation markers MKi67 and PCNA, change in the epithelial cells and impaired epithelial barrier, and upregulation of genes involved in pro-inflammatory and cellular death pathways [142].

**Figure 4:**
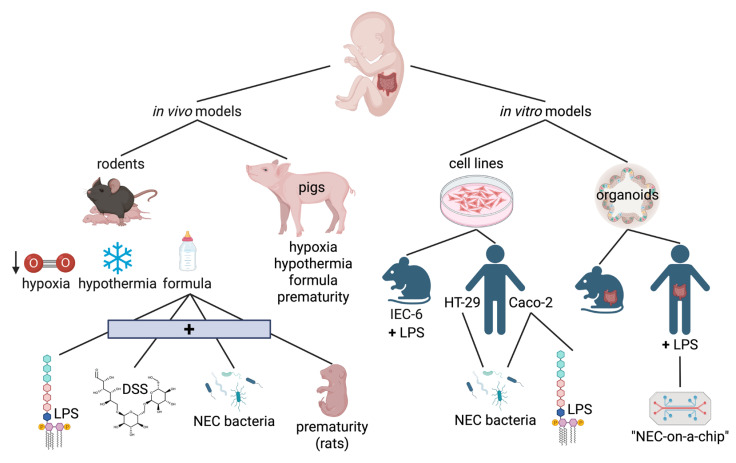
**Experimental research models of necrotizing enterocolitis.** In vivo models of NEC have primarily focused on using rodents and pigs in the past decade. NEC is induced in rodent models using a combination of hypoxia, hypothermia, and a formula that is supplemented with LPS, DSS, bacteria from patients with NEC, and/or premature rodents (rats only). In vitro models of NEC have taken advantage of various cell lines and, more recently, advanced into organoids. Cell lines include the IEC-6 line from rats, as well as the HT-29 and Caco-2 lines from humans. NEC is induced in these cell lines using either LPS or NEC-associated bacteria. Finally, intestinal organoids have been derived from either neonatal mouse pups with NEC or human preterm infants with NEC. Enteroids from infants are challenged with LPS to enhance the inflammatory effect and have been further cultured with a dysbiotic NEC microbiome in a “NEC-on-a-chip” microfluidic model. Created in BioRender. Sha, C. (2025) https://BioRender.com/ki85l7w.

In conclusion, both in vivo and in vitro models of NEC, summarized in Figure 4, are foundational to the study of this disease in a translational research setting. Animal models enable the analysis of NEC in a whole-body system, which is crucial for understanding the long-term consequences of the disease. The leading theory on the pathogenesis of NEC involves systemic immune responses and inflammation. Piglets are arguably more representative of human NEC due to their similarities in their gastrointestinal system and size, but they are much more difficult and costly to maintain compared to rodents. While rodent models have yielded numerous vital findings regarding NEC, one concern is that they often require formula supplementation with enteric bacteria, osmotic agents such as DSS, or exposure to hypoxia/hypothermia. Formula feeding alone is a significant risk factor for human NEC, raising questions about the direct translatability of rodent models. For the existing in vitro models, their greatest strength is the ability to sample directly from human NEC patients; however, they cannot study the systemic effects of the disease. In general, these models are constantly evolving to more closely mimic all the complexities of NEC as it presents in human patients. The ultimate goal of these models is to discover and test novel therapies that can improve disease prognosis and quality of life for these patients.

## 6. Current Challenges and Future Directions

### 6.1. Many Directions, One Goal…

Although NEC was initially described in the 1950s, it remains a vexing clinical problem for neonatologists and surgeons and continues to result in heart-wrenching situations for the parents and infants that suffer from it [143]. Through the efforts of organizations, such as the NEC Society, healthcare providers, and scientists, society collaborations and non-profit organizations there have been significant strides in understanding the pathophysiology of NEC. However, as shown in this review, much remains to be done in the diagnosis, treatment and prevention of this disease.

#### 6.1.1. Diagnosis and Prognosis

Bell’s classification and its modifications continue to be used in diagnosing and in guiding the treatment of NEC. Besides clinical judgment and abdominal X-ray findings, there are no other standard tests available for diagnosis and no imaging modality has replaced the abdominal X-ray. Faingold et al. described ultrasound criteria for defining NEC; however, due to operator dependence and institutional experience, as well as lack of standardized sizes for infants of various ages, ultrasound imaging has not become a standard adjunct for care [144]. Near-infrared spectroscopy has also been proposed as another modality for diagnosing and assessing bowel viability in NEC but it has not been widely adopted [145,146]. As described, many promising plasma and stool biomarkers have been studied. However, further research is required to validate and confirm their ability to prognosticate NEC. Large collaborative biobanks hold promise in organizing and aiding the identification of biomarkers [147]. Although there are three defined categories of NEC severity, NEC exists on a spectrum. Therefore, further tools, in terms of biomarkers and clinical tests, that can more accurately diagnose and predict the clinical course of patients, would greatly assist in guiding NEC care [148].

#### 6.1.2. Treatment and Management

There is a consensus on the initial management of NEC and absolute indications for surgery (Figure 2); however, the details and nuances of medical and surgical management of NEC are a constant source of discussion between neonatologists and pediatric surgeons [149]. Although trials have helped identify risk factors for peritoneal drainage, surgery, and timing, each patient presents their own unique confounding considerations. Furthermore, once patients have stabilized, management protocols, including medical adjuncts (such as supplements), antibiotic therapy length, and resumption of feeds, are an active area of clinical research [3,150]. Finally, a drug targeting a pathway unique to NEC remains out of reach, if even possible, considering the massive inflammatory cascade that is induced. Although the biochemical pathways underlying NEC pathology are starting to become characterized, how these pathways can be modulated or blocked to prevent the progression of NEC remains an active area of research utilizing both in vivo and in vitro models.

#### 6.1.3. Prevention

NEC is not only a disease that needs to be prevented, but its long-term sequelae also require prevention. The greatest risk factor for NEC remains prematurity and low birthweight [151]; research on how to prevent NEC in this vulnerable population is a major focus of clinical research. What supplements or microbiome modulators, in addition to breast milk, can prevent NEC? Multiple studies and systematic reviews have shown moderate evidence demonstrating that probiotic use decreases NEC [152,153,154]. In 2023, however, the US Federal Drug Administration (FDA) advised against the routine use of probiotics in preterm infants, especially those <1000 grams weight, because of documented sepsis mortality from probiotic supplementation in an infant. While trials on probiotics continue outside the United States, the FDA and other stakeholders have begun to discuss this issue and weigh the risk of sepsis against the benefits of probiotics for NEC [31,152,155,156,157].

Long-term morbidity of neonatal NEC includes gut morbidity, such as short bowel syndrome, gut dysmotility, and nutritional deficiencies. Furthermore, neurodevelopmental and cognitive delays have significant functional consequences for patients and their families; the mechanisms underlying these effects are poorly understood [158,159]. By understanding the processes underlying these complications, future treatments may help prevent them from occurring in the first place.

### 6.2. Future: Collaboration and Clinical Trials

Fortunately, infants with NEC today have a greater chance of survival than those described in the early case series of the 1950s due to advances in antibiotics, parenteral nutrition, surgery, and critical care [160]. Through the contributions of clinicians, scientists, and patient families, multi-institutional clinical studies on NEC management and treatment have built upon the basic science foundation of both in vivo and in vitro models of NEC. Due to the success of NEC treatment, another aspect of NEC research and therapy is its impact on patients’ and their families’ quality of life and mental health [19].

Indeed, NEC research has reached a critical mass, where breakthroughs are now possible. Through continued collaboration between clinicians, scientists, and patients’ families, and institutional or governmental support [161]. Someday, NEC may no longer be the dreaded diagnosis in the NICU.

## Figures and Tables

**Table 1:** Clinical staging of NEC based on physical and radiological signs (modified from [16]).

STAGE	SYSTEMIC SIGNS	INTESTINAL SIGNS	RADIOLOGIC SIGNS
**IA—** **SUSPECTED NEC**	Temperature instability, apnea, bradycardia, lethargy	Elevated pre-gavage residuals, mild abdominal distention, emesis, guaiac-positive stool	Normal or intestinal dilation, mild ileus
**IB—** **SUSPECTED NEC**	Same as IA	Bright red blood from rectum	Same as IA
**IIA—** **DEFINITE NEC,** **MILDLY ILL**	Same as IA	Same as above, plus absent bowel sounds, +/− abdominal tenderness	Intestinal dilation, ileus, pneumatosis intestinalis
**IIB—** **DEFINITE NEC, MODERATELY ILL**	Same as IA, plus mild metabolic acidosis, mild thrombocytopenia	Same as above, plus absent bowel sounds, definite abdominal tenderness, +/− abdominal cellulitis or right lower quadrant mass	Same as IIA, plus portal vein gas, +/− ascites
**IIIA—** **ADVANCED NEC, SEVERELY Ill, BOWEL INTACT**	Same as IIB, plus hypotension, bradycardia, severe apnea, combined respiratory and metabolic acidosis, disseminated intravascular coagulation, neutropenia	Same as above, plus signs of generalized peritonitis, marked tenderness, and distention of abdomen	Same as IIB, plus definite ascites
**IIIB—** **ADVANCED NEC, SEVERELY Ill, BOWEL PERFORATED**	Same as IIIA	Same as IIIA	Same as IIB, plus pneumoperitoneum

**Table 2:** Clinical trials.

NCT Identifier	Category (Type of Treatment)	NEC Outcome	Maternal, Paternal, or Infant-focused	Study Status	Study Results
NCT00252681	Surgical	NEC—PRIMARY OUTCOME	Infant	COMPLETED	NO
NCT00059449	Observational	NEC—PRIMARY OUTCOME	Infant	COMPLETED	NO
NCT01029353	Surgical	NEC—PRIMARY OUTCOME	Infant	COMPLETED	YES
NCT00707785	Drug Therapy	NEC—PRIMARY OUTCOME	Infant	COMPLETED	NO
NCT00005888	Dietary Supplement	NEC—PRIMARY OUTCOME	Infant	COMPLETED	NO
NCT02741648	Observational	NEC—PRIMARY OUTCOME	Infant	UNKNOWN	NO
NCT01106209	In vitro study	NEC—PRIMARY OUTCOME	Infant	TERMINATED	YES
NCT01222585	Antimicrobial Therapy	NEC—PRIMARY OUTCOME	Infant	COMPLETED	YES
NCT00747851	In vitro study	NEC—PRIMARY OUTCOME	Infant	UNKNOWN	NO
NCT01223261	Observational	NEC—PRIMARY OUTCOME	Infant	COMPLETED	NO
NCT02784821	Antimicrobial Therapy	NEC—PRIMARY OUTCOME	Infant	COMPLETED	YES
NCT00392977	Dietary Supplement	NEC—PRIMARY OUTCOME	Infant	COMPLETED	NO
NCT05535361	Device	NEC—OTHER OUTCOME	Infant	RECRUITING	NO
NCT03174301	Observational	NEC—SECONDARY OUTCOME	Infant	COMPLETED	NO
NCT00009646	Drug Therapy	NEC—SECONDARY OUTCOME	Infant	COMPLETED	NO
NCT01576003	Drug Therapy	NEC—SECONDARY OUTCOME	Infant	COMPLETED	YES
NCT01534481	Dietary Supplement	NEC—SECONDARY OUTCOME	Infant	COMPLETED	YES
NCT00339235	Observational	NEC—SECONDARY OUTCOME	Maternal	COMPLETED	NO
NCT03115463	Non-surgical	NEC—SECONDARY OUTCOME	Infant	COMPLETED	NO
NCT01473264	Drug Therapy	NEC—PRIMARY OUTCOME	Infant	COMPLETED	NO
NCT00955487	Non-surgical	NEC—SECONDARY OUTCOME	Infant	COMPLETED	YES
NCT01203475	Observational	NEC—PRIMARY OUTCOME	Infant	COMPLETED	NO
NCT00840983	Non-surgical	NEC—PRIMARY OUTCOME	Infant	COMPLETED	NO
NCT03997266	Antimicrobial Therapy	NEC—PRIMARY OUTCOME	Infant	RECRUITING	NO
NCT00734539	Antimicrobial Therapy	NEC—SECONDARY OUTCOME	Infant	COMPLETED	YES
NCT00114543	Non-surgical	NEC—SECONDARY OUTCOME	Infant	COMPLETED	NO
NCT03169881	Drug Therapy	NEC—SECONDARY OUTCOME	Infant	ACTIVE NOT RECRUITING	YES
NCT00005775	Drug Therapy	NEC—SECONDARY OUTCOME	Infant	COMPLETED	NO
NCT00809055	Drug Therapy	NEC—SECONDARY OUTCOME	Infant	COMPLETED	YES
NCT02451228	Drug Therapy	NEC—SECONDARY OUTCOME	Maternal	COMPLETED	NO
NCT03456336	Drug Therapy	NEC—SECONDARY OUTCOME	Infant	ACTIVE NOT RECRUITING	NO
NCT03728608	Non-surgical	NEC—SECONDARY OUTCOME	Infant	COMPLETED	YES
NCT02299414	Drug Therapy	NEC—SECONDARY OUTCOME	Maternal	COMPLETED	YES
NCT00615550	Drug Therapy	NEC—SECONDARY OUTCOME	Maternal	COMPLETED	YES
NCT05676476	Drug Therapy	NEC—SECONDARY OUTCOME	Infant	RECRUITING	NO
NCT04325308	Dietary Supplement	NEC—SECONDARY OUTCOME	Infant	ACTIVE NOT RECRUITING	YES
NCT00005777	Non-surgical	NEC—SECONDARY OUTCOME	Infant	TERMINATED	NO
NCT00233324	Non-surgical	NEC—SECONDARY OUTCOME	Infant	COMPLETED	YES
NCT01954056	Drug Therapy	NEC—SECONDARY OUTCOME	Infant	COMPLETED	YES
NCT05446272	Non-surgical	NEC—SECONDARY OUTCOME	Infant	RECRUITING	NO
NCT06153459	Non-surgical	NEC—PRIMARY OUTCOME	Infant	RECRUITING	NO
NCT00874393	Drug Therapy	NEC—SECONDARY OUTCOME	Infant	COMPLETED	NO
NCT03292731	Drug Therapy	NEC—SECONDARY OUTCOME	Maternal	TERMINATED	YES
NCT01866982	Non-surgical	NEC—SECONDARY OUTCOME	Infant	COMPLETED	NO
NCT06448780	Drug Therapy	NEC—SECONDARY OUTCOME	Infant	RECRUITING	NO
NCT01863043	Non-surgical	NEC—SECONDARY OUTCOME	Infant	COMPLETED	YES
NCT06605118	Antimicrobial Therapy	NEC—SECONDARY OUTCOME	Maternal	RECRUITING	NO
NCT01378273	Drug Therapy	NEC—SECONDARY OUTCOME	Infant	COMPLETED	YES
NCT00135707	Dietary Supplement	NEC—SECONDARY OUTCOME	Maternal	COMPLETED	YES
NCT01035697	Observational	NEC—SECONDARY OUTCOME	Infant	COMPLETED	NO
NCT00115934	Surgical	NEC—SECONDARY OUTCOME	Infant	COMPLETED	YES
NCT01827358	Antimicrobial Therapy	NEC—SECONDARY OUTCOME	Infant	COMPLETED	YES
NCT04519502	Observational	NEC—SECONDARY OUTCOME	Maternal	COMPLETED	NO
NCT00135902	Dietary Supplement	NEC—SECONDARY OUTCOME	Maternal	COMPLETED	NO
NCT03927833	Device	NEC—SECONDARY OUTCOME	Infant	ACTIVE NOT RECRUITING	NO
NCT01235546	Antimicrobial Therapy	NEC—PRIMARY OUTCOME	Maternal	COMPLETED	YES
NCT05459298	Dietary Supplement	NEC—SECONDARY OUTCOME	Infant	RECRUITING	NO
NCT06245057	Maternal Care	NEC—SECONDARY OUTCOME	Maternal	RECRUITING	NO
NCT01702805	Non-surgical	NEC—SECONDARY OUTCOME	Infant	ACTIVE NOT RECRUITING	YES
NCT06679855	Drug Therapy	NEC—SECONDARY OUTCOME	Infant	RECRUITING	NO
NCT06362798	Maternal Care	NEC—SECONDARY OUTCOME	Maternal	RECRUITING	NO
NCT06676904	Non-surgical	NEC—OTHER OUTCOME	Infant	RECRUITING	NO
NCT00388297	Drug Therapy	NEC—SECONDARY OUTCOME	Maternal	COMPLETED	YES
NCT00439374	Drug Therapy	NEC—SECONDARY OUTCOME	Maternal	TERMINATED	YES
NCT05380401	Dietary Supplement	NEC—OTHER OUTCOME	Infant	RECRUITING	NO
NCT00099164	Drug Therapy	NEC—SECONDARY OUTCOME	Maternal	COMPLETED	NO
NCT01376778	Drug Therapy	NEC—SECONDARY OUTCOME	Maternal	COMPLETED	YES
NCT01857310	Dietary Supplement	NEC—SECONDARY OUTCOME	Paternal	COMPLETED	YES
NCT02901626	Device	NEC—SECONDARY OUTCOME	Maternal	TERMINATED	YES
NCT03944512	Drug Therapy	NEC—SECONDARY OUTCOME	Maternal	TERMINATED	NO
NCT01954082	Drug Therapy	NEC—OTHER OUTCOME	Infant	TERMINATED	YES
NCT01778634	Antimicrobial Therapy	NEC—OTHER OUTCOME	Infant	COMPLETED	YES
NCT06878950	Dietary Supplement	NEC—OTHER OUTCOME	Infant	RECRUITING	NO
NCT01353313	Drug Therapy	NEC—SECONDARY OUTCOME	Infant	COMPLETED	YES
NCT04413097	Non-surgical	NEC—OTHER OUTCOME	Infant	ACTIVE NOT RECRUITING	NO
NCT06915428	Maternal Care	NEC—SECONDARY OUTCOME	Maternal	NOT YET RECRUITING	NO
NCT06980025	Drug Therapy	NEC—OTHER OUTCOME	Maternal	NOT YET RECRUITING	NO
NCT01203345	Drug Therapy	NEC—SECONDARY OUTCOME	Infant	COMPLETED	NO
NCT06855108	Drug Therapy	NEC—SECONDARY OUTCOME	Infant	NOT YET RECRUITING	NO
NCT00252681	Surgical	NEC—PRIMARY OUTCOME	Infant	COMPLETED	NO
NCT00059449	Observational	NEC—PRIMARY OUTCOME	Infant	COMPLETED	NO
NCT01029353	Surgical	NEC—PRIMARY OUTCOME	Infant	COMPLETED	YES
NCT00707785	Drug Therapy	NEC—PRIMARY OUTCOME	Infant	COMPLETED	NO
NCT00005888	Dietary Supplement	NEC—PRIMARY OUTCOME	Infant	COMPLETED	NO
NCT02741648	Observational	NEC—PRIMARY OUTCOME	Infant	UNKNOWN	NO
NCT01106209	In vitro study	NEC—PRIMARY OUTCOME	Infant	TERMINATED	YES
NCT00747851	In vitro study	NEC—PRIMARY OUTCOME	Infant	UNKNOWN	NO
NCT01223261	Observational	NEC—PRIMARY OUTCOME	Infant	COMPLETED	NO
NCT02784821	Antimicrobial Therapy	NEC—PRIMARY OUTCOME	Infant	COMPLETED	YES
NCT00392977	Dietary Supplement	NEC—PRIMARY OUTCOME	Infant	COMPLETED	NO
NCT05535361	Device	NEC—OTHER OUTCOME	Infant	RECRUITING	NO

**Table 3:** Experimental animal models of NEC.

Animal	Experimental NEC Protocol	Reference
Mouse	3-day protocol starting with 10-day-old C57BL/6 mice; Gavage feed hyperosmolar formula/Esbilac puppy milk replacer every 4 h; Hypoxia (100% N_2_ for 90 s) 3 times per day; Hypothermia (4 °C for 10 min) 3 times per day	[119]
Mouse	5-day protocol starting with 5-day-old BALB/c mice; Separate mice on night of Day 4 and fast overnight; Gavage with LPS 3 times per day and formula every 2 h (or overnight every 4 h); Hypoxia-reoxygenation-cold-shock cycle (5% O_2_ for 90 s, reoxygenate for 3 min, 4 °C for 15 min) 2 times per day	[120]
Mouse	3-day protocol starting with 3-day-old C57BL/6 mice; Gavage feed Esbilac puppy milk replacer with DSS every 3 h—mice were randomly assigned to a DSS concentration group (0%, 0.25%, 1%, 2%, or 3%)	[122]
Mouse	3-day protocol starting with 4-day-old C57BL/6 mice; Gavage feed NEC formula containing severe surgical NEC patient-derived enteric bacteria and LPS every 3 h; Hypoxia (95% N_2_ and 5% O_2_ for 10 min) 2 times per day	[124]
Mouse	4-day protocol starting with 7-day-old C57BL/6 mice; Gavage feed formula/Esbilac puppy milk replacer with enteric bacteria from infant with NEC 5 times per day; Hypoxia (95% N_2_ and 5% O_2_ for 10 min) 2 times per day	[125]
Mouse	4-day protocol starting with 5-day-old C57BL/6 mice; Gavage feed formula 3 times per day; Oral administration of LPS (4 mg/kg/day) with formula on days 1 and 2; Hypoxia (5% O_2_ for 10 min) 3 times per day	[126]
Rat	3-day protocol starting with 1-day-old SD rats; Gavage with 3% dextran sodium sulfate (DSS) dissolved in normal saline every 6 h or 4 times per day	[121]
Rat	4-day protocol starting with 20.5 days gestational age SD rats delivered prematurely via cesarean section; Gavage feed hypertonic, hypercaloric formula 5 times per day; Gavage 2 mg/kg LPS on the first day of life; Hypoxia (N_2_ for 90 s) and hypothermia (4 °C for 10 min) 3 times per day	[127]
Pig	8-day protocol starting with 106 days gestational age pigs delivered prematurely via cesarean section; Gavage feed increasing doses of human donor milk and decreasing doses of parenteral nutrition across 8 days	[129]
Pig	4-day protocol starting on 105–106 days gestational age pigs delivered prematurely via cesarean section; Gavage feed formula supplemented with enteric bacteria from an infant with surgical NEC every 3 h	[130]

## Data Availability

Not applicable.
